# Students' experiences of using ChatGPT for English language learning: a qualitative study in a Malaysian higher education institution

**DOI:** 10.3389/fpsyg.2026.1875727

**Published:** 2026-08-21

**Authors:** Nagaletchimee Annamalai, Mohamed Nasor, Arathai Din Eak, Amer Ayada Ayoub Alkubaisy

**Affiliations:** 1 Department of Languages and Culture, College of Humanities and Sciences, Ajman University, Ajman, United Arab Emirates; 2 Department of Biomedical Engineering, Ajman University, Ajman, United Arab Emirates; 3 School of Education, Humanities and Social Sciences, Wawasan Open University, Penang, Malaysia

**Keywords:** artificial intelligence, ChatGPT, English language learning, higher education, qualitative research

## Abstract

A qualitative thematic analysis was conducted with 25 students from a Malaysian institution to gain a deeper understanding of their motivation for using ChatGPT in English language learning. The study employed Self-Determination Theory (SDT) and the results demonstrated that ChatGPT has the potential to contribute positively to aspects related to English language competence, social connections, and independent learning processes. Therefore, ChatGPT emerges as a helpful resource for enhancing grammar, writing, and conversational tasks, allowing educators to prioritize higher order thinking abilities and intricate areas of language training. The results suggest that ChatGPT supports learners' autonomy, relatedness, and competence, which are key components of Self-Determination Theory (SDT). Nevertheless, the study also highlighted certain obstacles, such as ChatGPT's tendency to produce erroneous or nonsensical information and its restricted capacity to ensure the accuracy of content in language learning situations. These findings emphasize the need for focused efforts to tackle these difficulties. Educators must acknowledge the limitations of the tool and complement ChatGPT interactions with human interactions to provide a comprehensive and efficient language learning experience.

## 1 Introduction

Malaysian students have considerable obstacles in acquiring English language skills (Annamalai et al., 2023a,b). Their problems are related to rigid and traditional classroom teaching (Bhatt, 2021) and over-reliance on instructors in their teaching and learning activities (Zakaria and Sulaiman, 2024). Studies have documented that they lack proper English grammar knowledge and therefore are unable to construct appropriate sentences, which often leads to anxiety (Chigbu et al., 2023). Another language problem is that students lack the vocabulary knowledge and diverse vocabulary to express their ideas effectively (Khojasteh et al., 2023; Zakaria and Sulaiman, 2024). As a result, many learners struggle to communicate confidently in English and often perceive language learning as stressful and demanding. Such problems lead to low motivation in English language learning, which eventually hinders students' English language acquisition (Ag-Ahmad et al., 2025; Naeem et al., 2023). The Malaysian Education Blueprint (2013–2025) highlights the importance of integrating technology to improve English language skills, recognizing the capability of digital tools to address these difficulties (Ministry of Education Malaysia, 2013). Similarly, Olszewski and Crompton (2020) emphasized the importance of integrating technology into classroom instruction in shaping students' learning (Shen et al., 2023). The growing use of artificial intelligence (AI) in education has created new opportunities to support personalized, interactive language-learning experiences. ChatGPT, a language model utilizing Generative Pretrained Transformer technology, marked significant progress in the fields of artificial intelligence and natural language processing (Shen et al., 2023). ChatGPT's capacity to engage in meaningful, contextually appropriate conversations has garnered significant attention and enthusiasm (Annamalai et al., 2025; Foroughi et al., 2024). It has been utilized for many purposes such as generating content (Gao et al., 2024), providing tailored assistance (Tlili et al., 2023), improving writing abilities (Barrot, 2023), constructing Boolean questions for systematic reviews (Wang et al., 2021), and aiding in the creation of research papers (Macdonald et al., 2023). In English-language learning contexts, ChatGPT also enables learners to receive immediate feedback, practice writing skills, and engage in self-directed learning. (Tlili et al., 2023). In addition, the introduction of ChatGPT has prompted administrators to form task groups and organize institution-wide meetings to discuss its ramifications (Aydın and Karaarslan, 2022; Bollaert, 2025).

Studies have also documented the positive use of ChatGPT for motivation and English language learning. For example, Zare et al. (2025) reported that ChatGPT motivated students to enhance their argumentative writing. Similarly, Woo et al. (2024) found that English language learners were motivated to use ChatGPT for personalized learning and brainstorming support. Wang et al. (2024) also found that ChatGPT improved students' comprehension, independent learning, and increased academic efficiency and productivity. These findings suggest that ChatGPT has the potential to create a more engaging and learner-centered environment in English language classrooms (Annamalai and Nasor, 2025).

However, there have been criticisms of ChatGPT, particularly regarding critical thinking, ethics, plagiarism, and overreliance on the tool (Annamalai et al., 2025; Chinoracky and Stalmasekova, 2025; Zhang et al., 2026). These challenges highlight the need for more effective pedagogical approaches that better support AI's role in teaching and learning More structured activities with ChatGPT can help learners develop both critical awareness and responsible users (Annamalai et al., 2025). Therefore, it is important to examine how students meaningfully engage with ChatGPT in authentic educational settings (Kasneci et al., 2023).

Although a growing body of studies has investigated ChatGPT in language education, much of the existing literature has primarily focused on students' perceptions, attitudes, technology acceptance, learning outcomes and motivational benefits. These studies have demonstrated valuable findings on whether students find ChatGPT useful and how it may support language learning; nevertheless, they provide limited understanding of how learners experience and make sense of their interactions with ChatGPT in their learning activities. In particular, there remains a lack of studies investigating the subjective and meaning-making dimensions of learners' engagement with ChatGPT, including how they interpret ChatGPT's role in supporting their learning outcomes, how they negotiate challenges associated with its use, and how their experiences shape their motivation to learn English. Research on educational technology has emphasized that learners' experiences and interpretations are crucial for understanding how digital tools influence motivation, engagement, and learning behavior (Chiu, 2021; Ryan and Deci, 2020).

Furthermore, while previous studies have reported that ChatGPT can enhance motivation, little is known about the processes through which learners construct motivational meaning when interacting with AI-powered tools. Motivation is not merely an outcome of technology use but is also shaped by learners' personal experiences, interpretations, and social contexts. From a constructivist perspective, learners actively construct meaning through interactions with tools, previous experiences, and their learning environment, making qualitative exploration particularly valuable for understanding AI-assisted learning experiences (Vygotsky, 1978). Consequently, there is a pressing need for research that moves beyond measuring effectiveness and investigates the experiences through which learners understand, experience, and derive value from ChatGPT in English language learning.

A thematic qualitative study was employed to address the current gap because it seeks to understand how individuals experience a phenomenon and the meanings they attribute to those experiences. Qualitative inquiry is particularly appropriate when researchers aim to explore participants' subjective interpretations, experiences, and contextual meanings rather than merely measuring predefined variables (Creswell and Poth, 2018). Thematic analysis further enables researchers to identify recurring patterns across participants' narratives while preserving the richness and complexity of individual experiences (Braun and Clarke, 2021). By examining students' experiences, this study provides a deeper understanding of how ChatGPT influences learners' sense of autonomy, competence, and relatedness during English language learning. Such insights are important because they reveal dimensions of AI-assisted learning that may not be captured through surveys and technology acceptance models.

To achieve a meaningful and informed integration of ChatGPT into EFL writing instruction, an in-depth investigation of students' experiences is essential. Understanding how they perceive, engage with, and make sense of AI tools provides the foundation for designing pedagogically responsible and effective classroom practices. Therefore, instead of resisting and prohibiting ChatGPT in education, it would be advantageous to integrate it into the classroom as a strategy to help EFL learners improve their writing skills (Godwin-Jones, 2022; Zomer and Frankenberg-Garcia, 2021). Having said this, the current study examined the experiences of Malaysian language learners through the lens of Self-Determination Theory (SDT). According to SDT, learners are motivated when they can make their own decisions (autonomy), believe they are enhancing their skills (competence), and experience meaningful connections with others (relatedness) (Deci et al., 1991, 2017). The theory is particularly suitable for this study because it explains how motivation influences students' engagement, confidence, and persistence in language learning activities. SDT is particularly relevant to this study because motivation plays a crucial role in students' language learning. Moreover, SDT has been widely applied in technology-enhanced learning research because it provides a useful framework for examining how digital learning environments support or hinder learners' psychological needs and motivation (Chiu, 2021; Ryan and Deci, 2020). Furthermore, the theory provides valuable insights into how AI-assisted learning experiences may support students' psychological and educational needs in English language learning. The purpose of this initiative was to expand conversations, enhance future AI research in education, and provide information for policymaking, ultimately deepening our understanding of ChatGPT's capabilities and potential in educational environments.

The research question for this study is:

How does ChatGPT satisfy autonomy, competence, and relatedness?

The Self-Determination Theory, developed by Ryan and Deci (2020), explains the important social and contextual aspects necessary for human wellbeing and optimal performance. This statement suggests that humans have an intrinsic tendency to be proactive and inquisitive, with a natural inclination to investigate their environment, gain knowledge, and incorporate new experiences into a unified sense of self. This theory is applicable in a wide range of domains, including education. Ryan and Deci (2020) emphasized the importance of further investigating the potential of modern technology to motivate student learning and students‘ willingness to use technology for educational purposes. The approach has been widely and effectively used in both physical and virtual classrooms, producing positive outcomes (Annamalai and Nasor, 2025; Annamalai et al., 2023a; Bali et al., 2024; Chiu, 2021). The self-determination theory emphasizes that autonomy, competence, and relatedness are essential psychological needs for the development and overall wellbeing of an individual (Ryan and Deci, 2020). Although SDT is viewed as a traditional motivational theory, it remains highly relevant for examining learners' behavior in AI-enhanced learning environments (Annamalai et al., 2025; Xia et al., 2022). More importantly, SDT offers a useful framework for understanding students‘ use of ChatGPT, their experiences, and how these factors influence their motivation, agency, confidence, and sense of connection during language learning. While previous studies have largely focused on the effectiveness, acceptance, and educational benefits of ChatGPT, considerably less attention has been paid to understanding the subjective meanings learners attach to their interactions with AI technologies. Consequently, SDT provides a theoretically grounded lens through which to examine students' experiences with ChatGPT.

Autonomy, which refers to an individual's capacity to control their behaviors and make independent choices, is a crucial component in theories of motivation and learning (Ryan et al., 2021). In educational settings, autonomy-promoting environments empower learners to take control of their learning path, thereby increasing intrinsic motivation and active participation (Reeve, 2009). When contemplating the use of ChatGPT in English language instruction, autonomy empowers students to explore language acquisition independently, customizing their interactions with the technology to suit their individual needs and preferences.

However, autonomy in ChatGPT-mediated learning may develop complex problems rather than simply providing students with greater control. While ChatGPT can enhance self-directed and flexible learning pathways, it may also create a dependency on ChatGPT-generated assistance, indirectly reducing independent problem-solving (Bok and Cho, 2023). Thus, learners may simultaneously experience empowerment and reliance when engaging with ChatGPT (Jung et al., 2026). Examining how students negotiate these tensions requires exploring their experiences rather than solely measuring behavioral outcomes.

This study extends the concept of autonomy by examining the ChatGPT-mediated autonomy. In other words, the study investigated how learners negotiate meaning via adaptive feedback and personalized scaffolding. This perspective is supported by research indicating that personalized AI feedback can encourage learner agency and self-regulation when learners actively engage with the feedback and make informed decisions about their learning strategies (Lo, 2023). This introduces a nuanced interpretation of autonomy within AI-human interactions, which has been relatively underexplored in existing SDT research.

Relatedness refers to the feeling of connection, belonging, and social engagement that individuals have in their learning environment (Ryan and Deci, 2020). Nurturing significant connections and interpersonal exchanges is crucial for fostering engagement and cultivating a supportive educational community (Deci et al., 2017). Relatedness within ChatGPT-mediated learning needs further conceptual consideration. Traditionally, relatedness is defined as meaningful interactions with other individuals. In the context of ChatGPT, learners are not interacting with a human. ChatGPT interacts with learners, and this is viewed as content interaction (Annamalai and Nasor, 2025). Such experiences generate feelings of being accompanied in the absence of human interactions.

Rather than suggesting that ChatGPT replaces human interactions, this study explores whether learners experience psychological support through AI interactions and how these experiences shape their motivation for English language learning. Accordingly, the current study seeks to examine how students interpret and construct feelings of relatedness when engaging with ChatGPT, thereby contributing to ongoing discussions regarding the applicability of SDT within AI-enhanced educational environments.

Competence relates to individuals' effectiveness and capacity to successfully complete activities and accomplish desired results (Ryan and Deci, 2020). Providing chances for learners to feel capable and achieve success is crucial for maintaining motivation and fostering a growth mindset that believes in personal improvement. Scaffolding and feedback play a significant role in helping students develop their competency in ChatGPT-mediated language learning (Annamalai et al., 2025). Within the context of ChatGPT, competence may extend beyond improvements in grammar, vocabulary, and sentence structures. Learners may feel more capable because ChatGPT provides immediate guidance and corrective feedback (Van Horn, 2024). At the same time, questions remain regarding whether such experience reflects genuine language development or reliance on technology. Exploring how students make sense of their competence while using ChatGPT therefore represents an important area of inquiry that has received limited attention in existing studies (Jung et al., 2026).

In short, autonomy, relatedness, and competence are essential foundations of motivation and learning in educational settings. In the context of ChatGPT in English language learning, students may experience autonomy, competence, and relatedness needs in distinct ways. As such, this study examined not only SDT but also the scope of how these three needs are negotiated in human–AI interactions for English language learning.

## 2 Existing literature on ChatGPT-enhanced language learning

In recent years, studies on the use of ChatGPT in the educational context have gained momentum. For example, Ullah et al. (2026) employed a survey to gather participants' feedback on the usefulness and effectiveness of ChatGPT. The promising results suggest that ChatGPT is an effective tool for learning formal English. A qualitative study by Li et al. (2024) investigated how ChatGPT can assist and empower Chinese language learners. The results suggest that ChatGPT has potential as a supportive tool for Chinese language learners from economically disadvantaged backgrounds, thereby diminishing educational disparities and fostering fair access to language-learning opportunities. Nugroho et al. (2024) utilized ChatGPT to enhance students' writing abilities, finding that it facilitated translation, maintained academic precision, improved writing efficiency, and sparked ideas.

Similarly, Su et al. (2023) incorporated ChatGPT into a course on argumentative writing to help students structure outlines, craft content, refine language, and reflect on their work. In a study by Yan (2023), students showed a favorable disposition toward ChatGPT in L2 writing. They appreciated ChatGPT's prompt responses, rich contextual information, and diverse text formats. The students viewed ChatGPT as a valuable resource for L2 writing.

Although these studies provide valuable evidence regarding the educational potential of ChatGPT, they predominantly examine outcomes, perceptions, acceptance, and instructional effectiveness. Consequently, less is known about how learners subjectively experience using ChatGPT and how they construct motivational meanings from their interactions with the technology. In particular, there remains limited qualitative study exploring how learners experience autonomy, competence, and relatedness while engaging with ChatGPT for English language learning. This gap is significant because motivational experiences are not merely outcomes of technology use but are actively interpreted and negotiated by learners within specific educational and cultural contexts.

## 3 Methodology

This study adopted a qualitative thematic inquiry to investigate learners' experiences of employing ChatGPT in English language learning. Qualitative thematic analysis aims to develop an in-depth understanding of learners' engagement with ChatGPT and how they experience its influence in aspects related to competence, relatedness, and autonomy in English language learning. The study was guided by Self-Determination Theory (SDT) to explore students' experience related to competence, relatedness and autonomy. Rather than seeking the universal essence of an experience, the study focused on identifying recurring themes that reflected how students understood and utilized ChatGPT in their English language learning.

Although other sources, such as observation or system data, could offer behavioral insights, they would not reveal the depth of participants' perspectives and interpretations regarding their use of ChatGPT. Nevertheless, rigor was assured through methodological consistency, reflexivity, member checking, and peer debriefing, thereby enhancing the trustworthiness of the findings.

## 4 Participants

A sample of 25 undergraduate students enrolled in a public Malaysian university were employed in this study. Although 28 students were initially considered for participation, only 25 students were ultimately selected until data saturation was achieved (Howitt, 2012).

Purposive sampling was employed, as recommended by Patton (2002), to gather in-depth information from participants. The purposive selection of participants was guided by the principle of information richness, which holds that individuals with substantial experience using ChatGPT for English learning are better able to provide detailed descriptions of the phenomenon under investigation. Participants were selected based on predetermined inclusion criteria to ensure that they could provide information-rich accounts of using ChatGPT for English language learning. Participants were (a) students enrolled in the General English language course during the second semester of 2025; (b) have actively employed ChatGPT to support English language learning activities, including reading, writing, listening, speaking, grammar, or vocabulary tasks, for a minimum period of 5 months; and (c) demonstrate willingness to reflect on and discuss their experiences in detail during the interview process. These criteria were established to ensure that participants had sufficient exposure to ChatGPT and could meaningfully articulate their experiences. Participants ranged in age from 19 to 23 years and represented disciplines including business, engineering, information technology, and social sciences. Participants were also students who achieved Band 2 and Band 3 in the Malaysian University English Test, considered low-motivation students. These bands indicate relatively limited English proficiency, with Band 2 representing basic users and Band 3 representing modest users. Students within these proficiency levels often encounter greater challenges in English language learning and may require additional support and learning strategies. Consequently, they were considered suitable participants for exploring how ChatGPT contributed to their motivation and engagement in English learning. All the students were enrolled in the General English language course during the second semester of 2025. They were informed about the study's purpose and assured of confidentiality and anonymity. An email invitation was sent to all undergraduate students enrolled in the English course (focused on English proficiency). Students who expressed interest were asked to complete a screening form indicating the duration, frequency, and purpose of their ChatGPT use. Only those who met the inclusion criteria were invited to participate in the interviews. To ensure privacy, each student was assigned a pseudonym, such as G1, G2, and G3, etc. The instructor who conducted the study has a degree in Teaching English as a Second Language and has been teaching for 5 years. All procedures performed in this study adhere to the ethical standards of the 1964 Helsinki Declaration and its later amendments or comparable ethical standards. Therefore, this study was reviewed and granted exemption from ethical approval by Ajman University (Reference number: H-F-H-3—A) on 31 August 2023.

## 5 Research procedure

Instructors briefed the participants on the study's purpose and introduced ChatGPT 3.5 and ChatGPT 4.0. However, most students were familiar with ChatGPT but did not utilize it to complete the tasks and assignments given to them for their English proficiency course. In this study, the university permits educators to utilize current technology, including ChatGPT, in an ethical manner while employing effective teaching strategies. Participants were allowed to choose between ChatGPT 3.5 and ChatGPT 4.0 to support their English language learning activities throughout the semester. The 5-month period corresponded to the academic semester and represented sustained engagement with ChatGPT prior to the interviews. During this period, students used ChatGPT voluntarily to assist with course-related activities, including completing writing tasks, improving grammar and vocabulary, generating ideas for assignments, practicing speaking and listening activities, and obtaining feedback on their English language performance. For the purpose of participant selection, “use of ChatGPT” was defined as engaging with the platform at least twice a week for English learning-related purposes over the 5-month semester. Information regarding frequency and purpose of use was obtained through a preliminary screening questionnaire and verified during the interviews. Participants were asked to describe how they incorporated ChatGPT into their learning routines, enabling the researchers to confirm that they had sustained and meaningful experience with the tool. This procedure ensured that participants possessed sufficient exposure to ChatGPT to provide detailed reflections on their experiences.

## 6 Data collection

Interview questions were crafted to understand students' experiences with ChatGPT for English learning. These questions were derived from existing studies and literature reviews and validated by two experts in the fields of multimedia and education. The questions were also tested on two non-participants to ensure they were clear and unambiguous. The interviews followed an extensive, informal, and interactive procedure, characterized by open-ended comments and questions, as described by (Moustakas, 1994). The interview lasted 30–45 min for each participant. The interviews were recorded and transcribed verbatim. When probing is needed, questions like how” and “why” are used for better understanding and clarification.

The following practices suggested by Fisher and Frey (2014) were considered in this study:

- Listen more and talk less, as listening is the most important part of interviewing
- Follow up on what participants say and ask questions when you do not understand
- Avoid leading questions
- Do not interrupt. Learn how to wait
- Keep participants focused and ask for concrete details
- Tolerate silence. It means the participants are thinking
- Do not be judgmental about participants' views or beliefs; keep a neutral demeanor. Your purpose is to learn about others' perspectives, whether you agree with them or not.

The interview questions are:

- Can you describe your experience using ChatGPT for your English learning?
- Do you feel more controlled in your learning activities with the use of ChatGPT for English language learning?
- What do you think about interaction with ChatGPT?
- Do you feel supported when using ChatGPT, or does it make learning feel more isolated?

The interviewer employed several probing strategies to encourage participants to elaborate on their experiences, perceptions, emotions, and reflections regarding their use of ChatGPT for English language learning. These strategies included:

- Clarification probes—Could you explain what you mean by that?
- Elaboration probes—Can you tell me more about that experience?
- Feeling probes—How did that make you feel?
- Reflection probes—Why do you think this experience was significant for you?

## 7 Data analysis

The interview data were analyzed using Braun and Clarke (2021) thematic analysis method. The process involved several steps, beginning with the first author's extensive reading of transcripts and coding notes to become familiar with the dataset. Initial codes were then manually generated by carefully examining line-by-line transcripts. Subsequently, all codes were compiled, and those with similar concepts were grouped into categories. This phase shifted the focus from individual codes to broader themes, organizing different codes into potential themes and collating relevant coded data extracts within these identified themes. Two additional coders conducted the refinement and review of these themes. Deductive analyses were employed to categorize themes, including competence, relatedness, and autonomy, and inductive analysis was used. Both deductive and inductive coding approaches were employed. Deductive coding was based on the three dimensions of Self-Determination Theory (competence, relatedness, and autonomy), while inductive coding allowed new insights to emerge directly from participants' accounts. This combination enabled the study to remain theoretically informed while remaining open to unexpected patterns within the data. Themes were developed through an iterative and reflexive process in which the researchers actively engaged with the data, continuously refining interpretations and examining relationships among codes and themes. The final themes represented shared patterns of meaning across participants' experiences of using ChatGPT for English language learning.

## 8 Reflective practices

Reflexivity was considered throughout the study to acknowledge the researchers' dual roles as both course instructors and primary investigators. Reflective journaling was used before and after each interview to document the researcher's thoughts, emotions, and potential biases. These reflexive practices ensured that data interpretation remained grounded in participants' perspectives rather than in the researcher's instructional role. Several steps were taken to minimize bias and ensure the ethical integrity of the study. Firstly, participation was entirely voluntary, and students were informed that they could withdraw at any time during the study without affecting their grades. Secondly, students emphasized confidentiality, and the use of pseudonyms was intended to protect identities. Third, data collection and analysis were given to protect their identities. followed Moustakas (1994) qualitative approach.

## 9 Trustworthiness of qualitative data

In a qualitative study, it is essential to ensure trustworthiness at every stage of data collection (Mirhosseini, 2020). The researcher's honesty further enhances trustworthiness, as they are the primary instrument in a qualitative study. A detailed coding procedure, based on Braun and Clarke (2021), as illustrated above, was employed to ensure transparency and credibility. Transferability was achieved by describing the setting and participants. Two experts in technology and educational research also validated the interview questions.

Two coders coded the interview transcripts independently using a deductive approach. The transcripts were read multiple times to understand the significant statements that reflect their experience with ChatGPT English language learning. This was followed by grouping these statements into initial meaning units. After the grouping, both coders discussed their coding outputs. Discrepancies were resolved using a tiebreaker. To enhance reliability, inter-rater reliability was calculated using Cohen's Kappa coefficient,which yielded a value of 0.82, indicating a high level of agreement between coders. The final themes were established only after full agreement was achieved, ensuring that the coding process reflected the shared interpretation of the data rather than an individual bias. An audit trail was maintained, including coding notes, memos, and records of revisions made during the coding discussions. This documentation supports transparency in the analysis process and provides evidence of how consensus was reached among the researchers.

## 10 Findings

The following section presents findings from SDT, demonstrating how autonomy, competence, and relatedness shaped learners' experiences with ChatGPT in English language learning. In this study, the positive themes were categorized deductively based on competence, relatedness, and autonomy. In contrast, the negative themes were analyzed inductively, as they could not be clearly categorized into competence, relatedness, or autonomy. The negative themes were found to influence all three components of SDT.

### 10.1 Competence

These excerpts demonstrate how students experienced greater competence through their interactions with ChatGPT. Rather than merely receiving assistance from the tool, participants reported feeling more capable of handling English-language tasks, overcoming linguistic difficulties, and achieving learning goals independently. Their experiences reflect the SDT notion of competence, whereby individuals feel effective and confident in performing activities that are important to them.

G2 said that “ChatGPT is a helpful resource for my assignments in both English. For English courses, it aids me in essay writing by suggesting ideas, structuring content, and providing valuable editing and proofreading assistance.” One significant advantage of ChatGPT is that it provides learners with opportunities to engage in conversational practice, thereby enhancing their speaking proficiency, fluency, and pronunciation. For instance, the G7 said that “pairing with ChatGPT enhances speaking practice.” Similarly, the G21 highlighted that “students can practice forming responses, constructing sentences, and expressing ideas coherently.” G5 said that “ChatGPT can be used to practice making good sentences and delivering ideas coherently.

From a psychological aspect, their experiences contributed to learners' feelings of mastery and self-efficacy. Participants perceived themselves as becoming more fluent, articulate, and confident in expressing ideas in English. The opportunity to practice without fear of judgement allowed them to build confidence in their language abilities and to make progress in their learning.

The benefits of proficiency in English were highlighted from the perspective of translation. Participants expressed the advantages they gained from “translating from Malay language to English” (G7), highlighting it as a good practice that enhanced their linguistic abilities. G13 emphasized the importance of “translating when faced with challenging words.”

Moreover, ChatGPT is a valuable tool for overcoming cognitive barriers that arise while reading. Learners can use ChatGPT to obtain explanations, summaries, and content pertaining to new words or ideas. Participants felt less overwhelmed when confronting complex texts because they could seek immediate clarification. This experience reduced uncertainty and enabled them to maintain engagement with reading tasks, thereby strengthening their sense of competence as language learners.

Similarly, participants discussed how receiving feedback on their writing contributed to their learning. G1 stated that “I improved my writing abilities by obtaining comments on grammar, vocabulary, and concepts”. G7 explained that “in an English course, ChatGPT can provide topic suggestions, assist in generating ideas, and offer grammar checks to reduce errors.”

Participants experienced a growing sense of capability in producing written English. The ability to revise and refine their work fostered feelings of achievement and confidence, enabling them to view themselves as more competent writers. Overall, competence was experienced not merely through access to assistance but through learners' perceptions that they were becoming more effective, confident, and capable in navigating English language challenges.

### 10.2 Relatedness

Participants described experiences that reflected aspects of relatedness, particularly through feelings of being supported, understood, and accompanied during their learning process. Although ChatGPT is not a human interaction partner, learners often perceived the interaction as conversational and responsive, creating a sense of engagement that reduced feelings of isolation while learning.

G25 stated that “it offers valuable information and functions” as a versatile tool for language-related activities. G18 clarified that “conversing using ChatGPT resembles engaging in a conversation with an unfamiliar individual, where I express my problems and difficulties. These accounts suggest that participants imbued their interactions with ChatGPT with social meaning. The experience of expressing concerns, asking questions, and receiving responses created a perception of being listened to, which contributed to feelings of connectedness during learning activities.

G12 explained that it has helped me talk with people about certain topics. It helps me in my career for topics to talk about in new ways and for ways I can be prepared for a gathering.” Overall, participants acknowledged ChatGPT as a tool that facilitated their learning and problem-solving.

Participants opined that the interactions with ChatGPT enhanced their confidence in social communication by helping them prepare for conversations and interpersonal situations. As a result, learners felt better equipped socially and less anxious when engaging with others. Thus, relatedness was experienced not through genuine human connection but through a perceived sense of support that facilitated social participation and communication.

### 10.3 Autonomy

Participants reported a heightened sense of autonomy when they experienced self-direction and control over their learning processes. Rather than depending solely on lecturers or classmates, Participants felt that they are able to make decisions about how, when, and what they learned.

G22 explained that “when I have difficulties with grammar, ChatGPT provides me with a range of strategies, including utilizing grammar books and engaging in online exercises, to practice and improve my skills.” The availability of multiple learning options allowed participants to select strategies that aligned with their personal preferences and learning needs. This experience fostered a sense of ownership over their learning journey.

G3 claimed, “I am able to learn by myself based on feedback from ChatGPT,” while G7 said it “provides outputs tailored to my learning level”. Furthermore, G3 reached the conclusion that “ChatGPT offered personalized assistance and gave me space to learn on my own.” Participants experienced autonomy because they could independently regulate their learning, monitor their progress, and seek assistance when needed, without external pressure. The flexibility to engage with learning materials at their own pace strengthened their perception of self-directed learning.

G9 explained that “rather than blindly adopting the suggestions, I had the freedom to modify the idea based on my understanding”. This statement highlights learners' agency in evaluating, adapting, and making decisions about the information provided. Rather than passively accepting responses, participants exercised personal judgement, demonstrating a sense of volition that is central to SDT's conceptualization of autonomy.

Overall, autonomy was experienced through learners' perceptions of having control, choice, and responsibility in their English language learning rather than through the mere availability of technological assistance.

### 10.4 Negative experience

**Figure 1:**
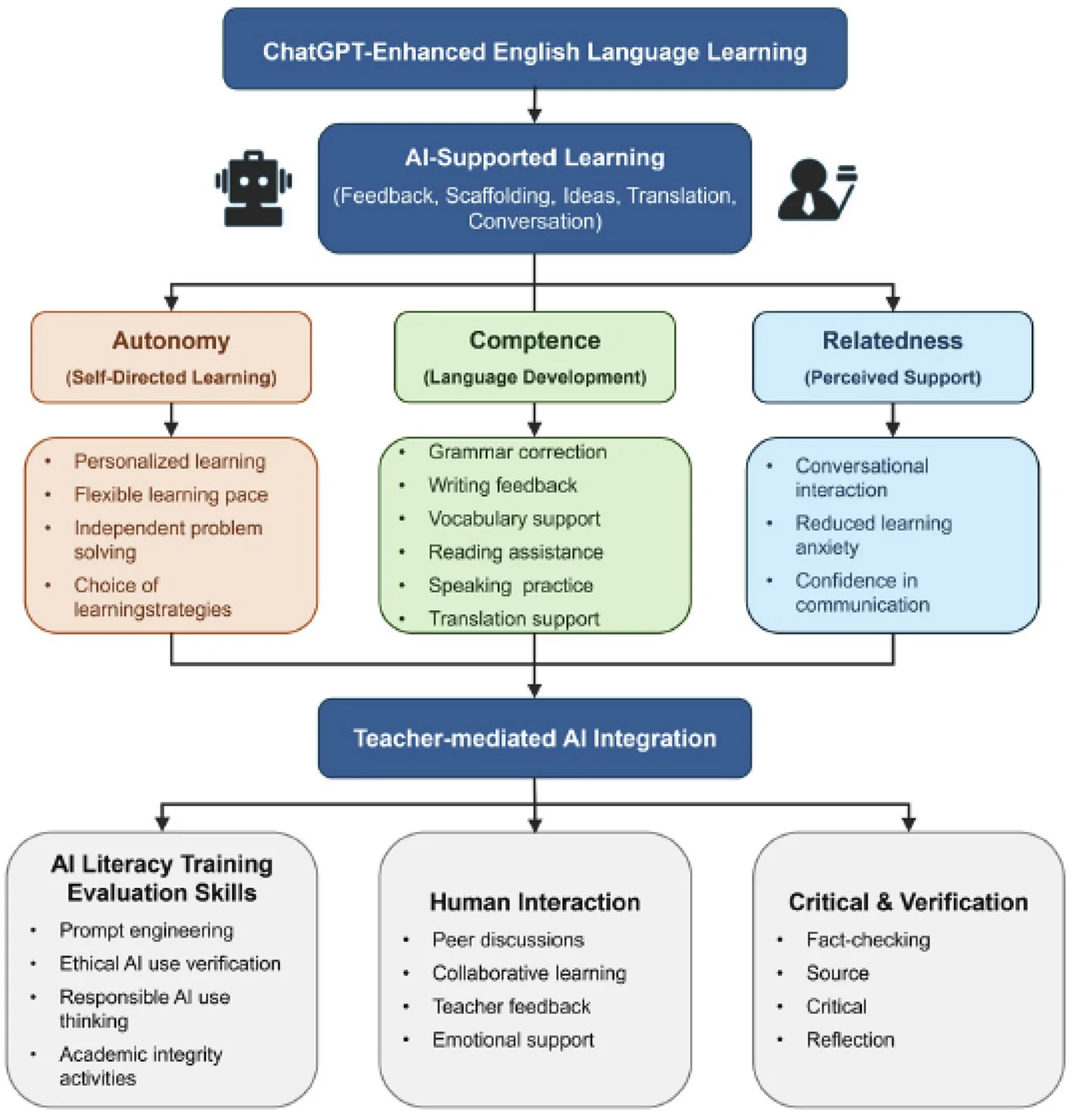
Pedagogical implication framework for ChatGPT-enhanced English language learning.

An important discovery was made regarding ChatGPT's limitations in real-world knowledge and its potential inability to consistently give context-specific responses during interactions, despite its value as a resource. A significant issue is that “at times ChatGPT can't offer appropriate responses during interaction” (G20). G7 found that it gives “inaccurate references.” Similarly, G11 emphasized that “ChatGPT produces incorrect results” (G11), which frequently led to misunderstandings and miscommunication. In addition, the respondents expressed frustration at encountering “illogical answers” (G2) during their learning path, which further hindered their understanding and advancement. Figure 1 illustrates the themes and subthemes related to competence.

Participants reported that inaccurate or misleading feedback reduces their confidence in the tool and requires extra effort to verify information. Consequently, these limitations constrained their sense of competence, as learners could not always trust the guidance received.

Nevertheless, they underscored that the final “responsibility for making a decision on the use of information from ChatGPT depends on the learner” (C2). This statement emphasizes that the participants realized the importance of employing critical thinking when using AI technologies, such as ChatGPT, to enhance English language proficiency.

In addition, participants emphasized the drawbacks of interacting with ChatGPT, such as “the lack of emotional connection and dependence on technology for language learning” (G7). In addition, users have observed that the drawbacks of engaging with ChatGPT include the “absence of emotional response and depending on technology for learning” (G5), emphasizing that “depending too much on ChatGPT and there is no human interaction” (G11). These limitations underscore the necessity for a well-rounded strategy that integrates human contact and emotional comprehension into the process of learning a language.

Students' experiences underscore that ChatGPT serves as an AI that scaffolds, not a substitute for, an instructor. These limitations reinforce the idea that instructors should facilitate the use of AI, as students need to distinguish between appropriate use and accurate information.

## 11 Discussion

The primary theoretical contribution of this research is to expand the current understanding of AI in education by establishing a connection with motivation driven by Self-Determination Theory (SDT). According to Self-Determination Theory (SDT), when students have their three basic needs met, their motivation becomes more internalized and driven by intrinsic variables within the activity itself (Ryan and Deci, 2020). The findings of this study have extended the Self-Determination Theory (SDT) by demonstrating how learners' basic psychological needs are reshaped through the use of an AI tool in language learning. SDT originally conceptualized autonomy, competence, and relatedness as human-centered constructs grounded in interpersonal and instructional contexts (Ryan and Deci, 2020). However, the current findings reveal a new AI-mediated motivational ecology in which needs are partly met through AI interaction rather than solely through instructor and peer support.

The findings demonstrate a form of AI-supported autonomy in which learners perceive greater flexibility and control over their learning activities through personalized feedback and self-directed exploration. Users can personalize their learning experiences according to their specific interests and needs, overcoming obstacles related to grammar with the help of ChatGPT. Learners demonstrate autonomy in their learning process by evaluating their progress and adapting their learning tactics according to the feedback provided by ChatGPT. Beyond merely correcting grammatical errors, participants described ChatGPT as reducing uncertainty during language production. The immediacy of feedback appeared to strengthen learners‘ confidence and willingness to experiment with language, suggesting that competence was experienced not simply as skill improvement but as a growing sense of linguistic capability. These results are consistent with a previous study that found ChatGPT improves learners' independence in acquiring language skills (Qi and Derakhshan, 2025; Godwin-Jones, 2022; Su et al., 2023).

Competence is evident when learners seem to progress from AI feedback that supports iterative learning and self-correction. It serves as a valuable resource for learners, providing assistance in essay writing through idea generation, editing support, translation, and immediate grammar correction (Gao et al., 2024; Nugroho et al., 2024; Zare et al., 2025). The tools' ability to enhance writing skills by providing feedback and corrections, thereby simplifying the writing process and promoting linguistic accuracy, has been highlighted by several researchers (Annamalai et al., 2025; Zare et al., 2025). These positive experiences tend to motivate students (Mozer et al., 2019) and help individuals to improve their learning (Ryan et al., 2021). Overall, ChatGPT has the capacity to assist learners in improving their language competency by providing feedback on their language usage and performance throughout the learning process. It serves as a helpful tool for practicing foreign languages, as observed by Bin-Hady et al. (2025). ChatGPT functions as an assistant rather than a primary source for obtaining information regarding the learning of the English language (Li et al., 2024).

Participants described conversational interactions with ChatGPT in ways that resembled social exchanges. The interactive nature of engaging in conversational practice with ChatGPT fosters the development of fluency in speaking and improves pronunciation. The results emphasized that learners gain advantages from surmounting comprehension barriers when reading with the assistance of ChatGPT's explanations and summaries. Nevertheless, whether these experiences fulfill the psychological need for relatedness, as conceptualized within SDT, remains debatable because authentic relatedness traditionally involves human interactions. Therefore, ChatGPT may provide perceived communicative support rather than fully satisfying the need for relatedness.

Many language learners strongly relate to the recurring theme of dissatisfaction that arises from delivering erroneous output. Previous studies are consistent with the present findings, indicating that the use of ChatGPT often yields unreliable information (Annamalai et al., 2025; Chinoracky and Stalmasekova, 2025; Zhang et al., 2026). Lack of precision in expressing views can lead to misconceptions and miscommunications, ultimately hindering one's ability to properly engage in English discourse. Koubaa et al. (2023) state that while ChatGPT possesses extensive background knowledge, it faces limitations when discussing topics beyond its training dataset. Prior research has also demonstrated that ChatGPT struggled to comprehend complex concepts and exhibited inconsistencies in its output Farrokhnia et al., (2024). According to Hsu and Ching (2023), there is a concern about ChatGPT's ability to provide accurate, trustworthy information. ChatGPT's propagation of inaccurate information and references can mislead learners.

Overall, ChatGPT in the context of English language acquisition provides a comprehensive strategy that promotes proficiency, connection, and independence. ChatGPT serves as a catalyst for enhancing language skills, deepening understanding of social interactions, and implementing personalized learning methodologies, ultimately leading to greater competence and mastery. With ongoing technological advancements, the use of AI tools such as ChatGPT has the potential to completely transform the field of English language teaching. This will enable learners to enhance their linguistic skills and achieve a high level of proficiency.

### 11.1 Pedagogical implications

The results of this study have several educational consequences for teachers and policymakers. ChatGPT offers personalized feedback and assistance, enabling teachers to implement differentiated instruction more effectively. This allows educators to accommodate a wide range of learning requirements inside the classroom. ChatGPT serves as a supplementary tool for aiding grammar, writing, and conversational practice. This allows teachers to allocate their attention toward higher-order thinking skills and more intricate areas of language training.

ChatGPT enables teachers to foster students' autonomy in their learning. The individualized feedback and assistance provided by the AI can help students develop greater self-direction and autonomy in their educational endeavors. Teachers can utilize the data and insights offered by ChatGPT to monitor student development and adjust instructional strategies, thereby ensuring that students remain aligned with their learning objectives. Furthermore, it is crucial for educators to recognize the potential emotional detachment that may arise during interactions with ChatGPT. Integrating human factors into teaching, such as peer conversations and teacher-led interactions, can help alleviate this problem and offer a more well-rounded learning experience. Although ChatGPT can aid language acquisition, educators should also emphasize the importance of interpersonal communication for cultivating sophisticated, emotionally profound language abilities. Policymakers should consider incorporating AI tools, such as ChatGPT, into the English language curriculum. This integration has the potential to enhance conventional teaching approaches and provide supplementary support to both students and teachers. It is essential to provide teachers with training and professional development on effectively utilizing AI tools in the classroom. This ensures that teachers can utilize these technologies to enhance student learning outcomes.

Training should focus on prompt engineering skills, strategies for evaluating AI-generated responses, understanding ethical and privacy considerations, and integrating AI outputs into pedagogical decision-making. Instructors need practical skills in guiding learners' ethical use of ChatGPT, designing AI-supported learning tasks, and utilizing AI assistance in human interactions to balance the cognitive, emotional, and relational aspects of language learning. Figure 1 illustrates Pedagogical implication framework for ChatGPT-enhanced English language learning.

## 12 Limitations, future studies and conclusion

While there has been considerable research on the application of ChatGPT in education, the perspectives and experiences of learners themselves have often been overlooked. This study addressed the lack of in-depth qualitative research by providing an understanding of the experiences of Malaysian university students in utilizing ChatGPT for English language acquisition. The students expressed that their experiences were generally favorable, particularly in terms of competence, relatedness, and autonomy, but participants also stressed the importance of exercising caution in relying too heavily on information. They advised users to carefully evaluate responses and verify information from trustworthy sources as necessary. These findings contribute to the growing body of qualitative research by providing contextually grounded insights into how learners experience ChatGPT-mediated English language learning through the lens of Self-Determination Theory. Rather than seeking universal explanations, the study offers a nuanced understanding of learners' perceptions within a specific educational setting, thereby informing educators and researchers interested in AI-supported language learning in comparable contexts.

This study is subject to multiple constraints. As a qualitative inquiry conducted within a single Malaysian higher education institution, the findings should be interpreted within their specific educational and sociocultural context. The purpose of this study was not to achieve statistical generalization but to develop an in-depth understanding of learners experiences of using ChatGPT for English language learning. Consequently, the transferability of the findings should be considered in relation to educational settings that share similar learner characteristics, institutional contexts, and pedagogical practices. The participants‘ experiences were explored over a single academic semester, which may not fully capture how learners' perceptions, motivations, and patterns of AI use evolve over time. Future research could build upon these findings by conducting comparative qualitative studies across different higher education institutions, disciplines, or cultural contexts to examine how contextual factors shape learners‘ experiences with AI-mediated language learning. Longitudinal qualitative research would also provide valuable insights into how learners' autonomy, competence, and relatedness develop as they gain sustained experience with ChatGPT. In addition, future studies may further refine theoretical explanations by exploring the interaction between Self-Determination Theory and other complementary perspectives in understanding AI-supported language learning. Mixed-methods and quantitative studies may complement these qualitative findings by examining broader patterns across larger populations; however, such approaches should be viewed as extending rather than replacing the contextual insights generated through qualitative inquiry.

Furthermore, Internet stability may likely influence participants engagement with the tool. Such a challenge can be considered a contextual limitation that can influence the interpretation of the findings. As with all qualitative research, the findings are inevitably shaped by the interpretive role of the researchers. The researchers‘ prior knowledge, experiences, and perspectives regarding ChatGPT may have influenced the interpretation of participants' accounts despite the use of reflexive practices throughout the research process. Although measures such as continuous reflection, collaborative discussions, and systematic thematic analysis were employed to enhance trustworthiness, complete objectivity cannot be assumed. These interpretive considerations should therefore be viewed as inherent characteristics of qualitative inquiry rather than methodological weaknesses.

## Data Availability

The raw data supporting the conclusions of this article will be made available by the authors, without undue reservation.

## References

[B1] Ag-Ahmad N. Mohamed A. T. F. S. Majilang D. B. (2025). Voices from ESL classrooms: overcoming challenges and enhancing English language teacher education in Malaysia. Stud. Engl. Lang. Educ. 12, 328–345. doi: 10.24815/siele.v12i1.39831

[B2] Annamalai N. Bervell B. Mireku D. O. Andoh R. P. K. (2025). Artificial intelligence in higher education: modelling students' motivation for continuous use of ChatGPT based on a modified self-determination theory. Comput. Educ. Artif. Intell. 8:100346. doi: 10.1016/j.caeai.2024.100346

[B3] Annamalai N. Eltahir M. E. Zyoud S. H. Soundrarajan D. Zakarneh B. Al Salhi N. R. (2023a). Exploring English language learning via Chabot: a case study from a self-determination theory perspective. Comput. Educ. Artif. Intell. 5:100148. doi: 10.1016/j.caeai.2023.100148

[B4] Annamalai N. Nasor M. (2025). Exploring ChatGPT in education: unveiling learners' experiences through the lens of self-determination theory. Smart Learn. Environ. 12:59. doi: 10.1186/s40561-025-00393-2

[B5] Annamalai N. Uthayakumaran A. Zyoud S. H. (2023b). High school teachers' perception of AR and VR in English language teaching and learning activities: a developing country perspective. Educ. Inf. Technol. 28, 3117–3143. doi: 10.1007/s10639-022-11275-2

[B6] Aydın Ö. Karaarslan E. (2022). “OpenAI ChatGPT generated literature review: digital twin in healthcare,” in Emerging Computer Technologies 2, ed. Ö. Aydın (İzmir: İzmir Akademi Derneği), 22–31. doi: 10.2139/ssrn.4308687

[B7] Bali S. Suwandi E. Chen T. C. Lin C. Y. Liu M. C. (2024). Social influence, personal views, and behavioral intention in ChatGPT adoption. J. Comput. Inform. Syst. 1–12. doi: 10.1080/08874417.2024.2441758

[B8] Barrot J. S. (2023). Using ChatGPT for second language writing: pitfalls and potentials. Assess. Writ. 57:100745. doi: 10.1016/j.asw.2023.100745

[B9] Bhatt S. P. (2021). Self-directed professional development: EFL teachers' understanding. Int. J. Language Literary Stud. 3, 196–208. doi: 10.36892/ijlls.v3i4.737

[B10] Bin-Hady W. R. A. Al-Kadi A. Hazaea A. Ali J. K. M. (2025). Exploring the dimensions of ChatGPT in English language learning: a global perspective. Libr. Hi Tech 43, 1315–1328. doi: 10.1108/LHT-05-2023-0200

[B11] Bok E. Cho Y. (2023). Examining Korean EFL college students' experiences and perceptions of using ChatGPT as a writing revision tool. J. Eng. Teach. Movie Media 24, 15–27. doi: 10.16875/stem.2023.24.4.15

[B12] Bollaert L. (2025). Artificial intelligence: objective or tool in the 21st-century higher education strategy and leadership?. Educ. Sci. 15:774. doi: 10.3390/educsci15060774

[B13] Braun V. Clarke V. (2021). One size fit all?What counts as quality practice in (reflexive) thematic analysis? Qual. Res. Psychol. 18,328–352. doi: 10.1080/14780887.2020.1769238

[B14] Chigbu G. U. Emelogu N. U. Egbe C. I. Okoyeukwu N. G. Eze K. O. Nwafor C. K. . (2023). Enhancing ESL students' academic achievement in expository essay writing using digital graphic organisers: a mixed-methods research. Heliyon 9:e15589. doi: 10.1016/j.heliyon.2023.e15589 37153431 PMC10160756

[B15] Chinoracky R. Stalmasekova N. (2025). Ethical problems in the use of artificial intelligence by university educators. Educ. Sci. 15:1322. doi: 10.3390/educsci15101322

[B16] Chiu T. K. F. (2021). Digital support for student engagement in blended learning based on self-determination theory. Comput. Human Behav. 124:106909. doi: 10.1016/j.chb.2021.106909

[B17] Chiu T. K. F. (2021b). Student engagement in K-12 online learning amid COVID-19: a qualitative approach from a self-determination theory perspective. Interact. Learn. Environ. doi: 10.1080/10494820.2021.1926289

[B18] Creswell J. W. Poth C. N. (2018). Qualitative Inquiry and Research Design: Choosing Among Five Approaches, 4th Edn. Thousand Oaks, CA: SAGE Publications.

[B19] Deci E. L. Olafsen A. H. Ryan R. M. (2017). Self-determination theory in work organizations: the state of a science. Annu. Rev. Organ. Psychol. Organ. Behav. 4, 19–43. doi: 10.1146/annurev-orgpsych-032516-113108

[B20] Deci E. L. Vallerand R. J. Pelletier L. G. Ryan R. M. (1991). Motivation and education: the self-determination perspective. Educ. Psychol. 26, 325–346. doi: 10.1080/00461520.1991.9653137

[B21] Farrokhnia M. Banihashem S. K. Noroozi O. Wals A. (2024). A SWOT analysis of ChatGPT: implications for educational practice and research. Innov. Educ. Teach. Int. 61, 460–474. doi: 10.1080/14703297.2023.2195846

[B22] Fisher D. Frey N. (2014). Checking for Understanding: Formative Assessment Techniques for Your Classroom. Alexandria, VA: ASCD.

[B23] Foroughi B. Senali M. G. Iranmanesh M. Khanfar A. Ghobakhloo M. Annamalai N. . (2024). Determinants of intention to use ChatGPT for educational purposes: findings from PLS-SEM and fsQCA. Int. J. Hum. Comput. Interact. 40, 4501–4520. doi: 10.1080/10447318.2023.2226495

[B24] Gao N. Zhao Z. Zeng Z. Zhang S. Weng D. Bao Y. (2024). GesGPT: speech gesture synthesis with text parsing from ChatGPT. IEEE Robot. Autom. Lett. 23, 2718–2725 doi: 10.1109/LRA.2024.3359544

[B25] Godwin-Jones R. (2022). Partnering with AI: intelligent writing assistance and instructed language learning. Lang. Learn. Technol. 26, 5–24. doi: 10.64152/10125/73474

[B26] Howitt D. (2012). “Using qualitative methods to research offenders and forensic patients,” in Research in Practice for Forensic Professionals, eds. K. Harrison, B. Rainey, and R. Jones (London: Routledge), 132–158.

[B27] Hsu Y. C. Ching Y. H. (2023). Generative artificial intelligence in education, part one: the dynamic frontier. TechTrends 67, 603–607. doi: 10.1007/s11528-023-00863-9

[B28] Jung J. Joo S. Park H. Han I. (2026). Challenges of integrating ChatGPT into EFL writing: an Activity Theory framework. Comput. Assist. Lang. Learn. 1–24. doi: 10.1080/09588221.2026.2617386

[B29] Kasneci E. Seßler K. Küchemann S. Bannert M. Dementieva D. Fischer F. Kasneci G. (2023). ChatGPT for good? On opportunities and challenges of large language models for education. Learn. Individ. Differ. 103:102274. doi: 10.1016/j.lindif.2023.102274

[B30] Khojasteh L. Karimian Z. Farahmandi A. Y. Nasiri E. Salehi N. (2023). E-content development of English language courses during COVID-19: a comprehensive analysis of students' satisfaction. J. Comput. Educ. 10, 107–133. doi: 10.1007/s40692-022-00224-0

[B31] Koubaa A. Boulila W. Ghouti L. Alzahem A. Latif S. (2023). Exploring ChatGPT capabilities and limitations: a survey. IEEE ACCESS 11:118698–118721. doi: 10.1109/ACCESS.2023.3326474

[B32] Li L. Ma Z. Fan L. Lee S. Yu H. Hemphill L. (2024). ChatGPT in education: a discourse analysis of worries and concerns on social media. Educ. Inf. Technol. 29, 10729–10762. doi: 10.1007/s10639-023-12256-9

[B33] Lo C. K. (2023). What is the impact of ChatGPT on education? A rapid review of the literature. Educ. Sci. 13:410. doi: 10.3390/educsci13040410

[B34] Macdonald C. Adeloye D. Sheikh A. Rudan I. (2023). Can ChatGPT draft a research article? An example of population-level vaccine effectiveness analysis. J. Glob. Health. 13:01003. doi: 10.7189/jogh.13.01003 36798998 PMC9936200

[B35] Ministry of Education Malaysia (2013). Malaysia Education Blueprint 2013–2025: Preschool to Post-Secondary Education. Kuala Lumpur: Kementerian Pendidikan Malaysia. 283

[B36] Mirhosseini S. A. (2020). Doing Qualitative Research in Language Education. Cham: Springer Nature. doi: 10.1007/978-3-030-56492-6

[B37] Moustakas C. (1994). Phenomenological Research Methods. Thousand Oaks, CA: Sage. doi: 10.4135/9781412995658

[B38] Mozer M. C. Wiseheart M. Novikoff T. P. (2019). Artificial intelligence to support human instruction. Proc. Nat. Acad. Sci. 116, 3953–3955. doi: 10.1073/pnas.1900370116 30782820 PMC6410845

[B39] Naeem M. Ozuem W. Howell K. Ranfagni S. (2023). A step-by-step process of thematic analysis to develop a conceptual model in qualitative research. Int. J. Qual. Methods. 22, 1–18. doi: 10.1177/16094069231205789

[B40] Nugroho A. D. Dwayne G. S. Arrazaqu K. I. Baihaqi M. N. Wibowo H. Z. A. Widyatami C. C. Araminta A. N. (2024). Implementasi AI ChatGPT Sebagai Alat Pendukung Pembelajaran Mahasiswa pada Prodi Sistem Informasi di Perguruan Tinggi Universitas Negeri Semarang. Jurnal Mediasi 3, 106–118.

[B41] Olszewski B. Crompton H. (2020). Educational technology conditions to support the development of digital age skills. Comput. Educ. 150:103849. doi: 10.1016/j.compedu.2020.103849

[B42] Patton M. Q. (2002). Two decades of developments in qualitative inquiry: a personal, experiential perspective. Qual. Soc. Work 1:261– doi: 10.1177/1473325002001003636

[B43] Qi S. Derakhshan A. (2025). Technology-based collaborative learning: EFL learners' social regulation and modifications in their academic emotions and academic performance. Educ. Inf. Technol. 30, 8611–8636. doi: 10.1007/s10639-024-13167-z

[B44] Reeve J. (2009). Why teachers adopt a controlling motivating style toward students and how they can become more autonomy supportive. Educ. Psychol 44, 159–175. doi: 10.1080/00461520903028990

[B45] Ryan R. M. Deci E. L. (2020). Intrinsic and extrinsic motivation from a selfdetermination theory perspective. Definitions, theory, practices, and future directions. Contemp. Educ. Psychol. 61:101860. doi: 10.1016/j.cedpsych.2020.101860

[B46] Ryan R. M. Deci E. L. Vansteenkiste M. Soenens B. (2021). Building a science of motivated persons: self-determination theory's empirical approach to human experience and the regulation of behavior. Motiv. Sci. 7:97. doi: 10.1037/mot0000194

[B47] Shen Y. Heacock L. Elias J. Hentel K. D. Reig B. Shih G. . (2023). ChatGPT and other large language models are double-edged swords. Radiology 307:e230163. doi: 10.1148/radiol.230163 36700838

[B48] Su Y. Lin Y. Lai C. (2023). Collaborating with ChatGPT in argumentative writing classrooms. Assess. Wri. 57:100752. doi: 10.1016/j.asw.2023.100752

[B49] Tlili A. Shehata B. Adarkwah M. A. Bozkurt A. Hickey D. T. Huang R. Agyemang B. (2023). What is the devil is my guardian angel: ChatGPT as a case study of using chatbots in education. Smart Learn. Environ. 10, 1–24. doi: 10.1186/s40561-023-00237-x

[B50] Ullah R. Shaikh M. S. Shahani N. Lone M. A. Fareed M. A. Zafar M. S. (2026). Comparing ChatGPT and dental students' performance in an introduction to dental anatomy examination: a cross-sectional study. Eur. J. Dent. 20, 287–294. doi: 10.1055/s-0045-1808254 40359999 PMC12890407

[B51] Van Horn K. R. (2024). ChatGPT in English language learning: exploring perceptions and promoting autonomy in a university EFL context. TESL-EJ Second 28:1. doi: 10.55593/ej.28109a8

[B52] Vygotsky L. S. (1978). Mind in Society: The Development of Higher Psychological Processes. Cambridge, MA: Harvard University Press.

[B53] Wang X. Wong Y. D. Liu F. Yuen K. F. (2021). A push–pull–mooring view on technology-dependent shopping under social distancing: when technology needs meet health concerns. Technol. Forecast. Soc. Change 173:121109. doi: 10.1016/j.techfore.2021.121109

[B54] Wang X. Zhong Y. Huang C. Huang X. (2024). ChatPRCS: a personalized support system for English reading comprehension based on ChatGPT. IEEE Trans. Learn. Technol. 17, 1722–1736. doi: 10.1109/TLT.2024.3405747

[B55] Woo D. J. Wang D. Guo K. Susanto H. (2024). Teaching EFL students to write with ChatGPT: students' motivation to learn, cognitive load, and satisfaction with the learning process. Educ. Inf. Technol. 29, 24963–24990. doi: 10.1007/s10639-024-12819-4

[B56] Xia Q. Chiu T. K. Lee M. Sanusi I. T. Dai Y. Chai C. S. (2022). A self-determination theory (SDT) design approach for inclusive and diverse artificial intelligence (AI) education. Comput. Educ. 189:104582. doi: 10.1016/j.compedu.2022.104582

[B57] Yan D. (2023). Impact of ChatGPT on learners in a L2 writing practicum: an exploratory investigation. Educ. Inf. Technol. 28, 13943–13967 doi: 10.1007/s10639-023-11742-4

[B58] Zakaria N. Sulaiman N. A. (2024). The needs analysis of ESL learners' expository writing challenges: perspectives of ESL teachers. Int. J. Acad. Res. Busin. Soc. Sci. 14, 322–335. doi: 10.6007/IJARBSS/v14-i5/21387

[B59] Zare J. Ranjbaran Madiseh F. Derakhshan A. (2025). Generative AI and English essay writing: Exploring the role of ChatGPT in enhancing learners' task engagement. Appl. Linguist. doi: 10.1093/applin/amaf045. [Epub ahead of print].

[B60] Zhang Y. Zhou T. Qiao H. Li T. (2026). Ethical issues in AI-generated texts: a systematic review and analysis. Int. J. Hum.-Comput. Interact. 42, 2586–2613. doi: 10.1080/10447318.2025.2530071

[B61] Zomer G. Frankenberg-Garcia A. (2021). “Beyond grammatical error correction: improving L1-influenced research writing in English using pre-trained encoder-decoder models,” in Findings of the Association for Computational Linguistics: EMNLP 2021, eds. M.-F. Moens, X. Huang, L. Specia, and S. W.-T. Yih (Association for Computational Linguistics), 2534–2540. doi: 10.18653/v1/2021.findings-emnlp.216

